# Associations of childhood experiences and methamphetamine use among Akha and Lahu hill tribe youths in northern Thailand: A cross-sectional study

**DOI:** 10.1371/journal.pone.0234923

**Published:** 2020-06-18

**Authors:** Tawatchai Apidechkul, Chalitar Chomchoei, Pilasinee Wongnuch, Ratipark Tamornpark, Panupong Upala, Fartima Yeemard, Marisa Poomiphak Na Nongkhai, Woottichai Nachaiwieng, Rachanee Sunsern

**Affiliations:** 1 Center of Excellence for The Hill Tribe Health Research, Mae Fah Luang University, Chiang Rai, Thailand; 2 School of Health Science, Mae Fah Luang University, Chiang Rai, Thailand; 3 Chulabhorn Royal Academy, Bangkok, Thailand; National University of Singapore, SINGAPORE

## Abstract

**Background:**

Methamphetamine (MA) is a commonly used substance among youths, particularly those who are living in poor economic conditions with low levels of education and who have had bad childhood experiences. The Akha and Lahu hill tribe youths living on the Thailand-Myanmar-Laos border are identified as the group most vulnerable to MA use in Thailand. The study aimed to estimate the prevalence of MA use and determine its associations with childhood experiences among Akha and Lahu youths aged 15–24 years in northern Thailand.

**Methods:**

A cross-sectional study was performed. Validated and sealed questionnaires were used to gather information from participants after obtaining the informed consent form. Questionnaires were completed by participants and their parents at home. Logistic regression was used to identify the associations between variables at the α = 0.05 level.

**Results:**

A total of 710 participants participated in the study: 54.2% were Akha, 52.5% were females, 50.6% were aged 15–17 years, and 11.4% did not have Thai identification card (ID) cards. The overall prevalence of MA use at least once among Akha and Lahu youths was 14.5%. After controlling for all potential confounding factors, 8 variables were found to be associated with MA use. Males had a greater chance of MA use than females (AOR = 4.75; 95% CI = 2.27–9.95). Participants aged 21–24 years had a greater chance of MA use than those aged 15–17 years (AOR = 2.51; 95% CI = 1.11–5.71). Those who had a family member who used MA had a greater chance of MA use than those who did not (AOR = 5.04; 95% CI = 1.66–15.32). Those who had been physically assaulted by a family member while aged 0–5 years had a greater chance of MA use than those who had not (AOR = 2.29; 95% CI = 1.02–5.12). Those who had been physically assaulted by a family member while aged 6–14 years had a greater chance of MA use than those who had not (AOR = 3.15; 95% CI = 1.32–7.54). Those who had a close friend who used alcohol had a greater chance of MA use than those who did not (AOR = 2.24; 95% CI = 1.24–4.72). Those who had a highly confident personality had a greater chance of MA use than those who did not (AOR = 2.35; 95% CI = 1.17–4.69), and those who smoked had a greater chance of MA use than those who did not (AOR = 8.27; 95% CI = 4.42–15.46).

**Conclusions:**

All relevant government and nongovernment agencies together with the Ministry of Public Health Thailand should address MA use among Akha and Lahu youths by properly developing a community health intervention that lowers risk of MA use by addressing family relationships, male youth behaviors, and focused on those individuals with a highly confident personality.

## Introduction

Methamphetamine (MA) has been widely recognized as the original factor contributing to several problems from individual health problems to social problems, including physical and mental health problems [1], poor family relationships [2], social problems [3], and interference with country economic growth [4]. Today, MA use is resulting in large social and economic problems globally [5]. Many communities have faced a severe stage of problems associated with MA use, particularly disruption of community economic growth [6]. The United Nations Office on Drugs and Crime (UNODC) reported that 5.6% (275 million people) of the global population aged 15–64 years used drugs at least once in 2016 [7]. In 2018, a study reported that MA was the second most commonly used illicit drug worldwide, and the Southeast Asia region was the most impacted region, including Thailand [8]. In 2019, the Department of Mental Health Ministry of Public Health, Thailand reported that there were 2.7 million Thai youths using MA [9]. Northern Thailand is considered a region of MA production and distribution due to sharing borders with Myanmar and Laos, which are recognized as the largest regions of MA production in the world [10]. Today, MA is becoming easily available in these areas due to the decrease in its price, particularly in border areas [11].

The most vulnerable group for MA use is the youth group [12]. Youths range in age from 15–24 years according to the definition of the United Nations [13]. Youths with poor socioeconomic status are reported as the group most vulnerable to MA use in Thailand, and this is particularly true among hill tribe youths [14]. The hill tribe is a group of people who have migrated into Thailand from southern China over several centuries [15] which is consisted of six main tribes: Akha, Lahu, Yao, Karen, Hmong, and Lisu [16]. The United Nations reported that most of the hill tribe people in Thailand lived below the poverty level in Thailand [17]. Akha is the largest group, followed by Lahu [16]. These two groups accounted for more than 70.0% [18] of the total hill tribe populations in Thailand, which was 3.5–4 million in 2018 [19]. Akha and Lahu have their own culture, language, and lifestyle pattern, including attitudes and perceptions toward drugs. Most Akha and Lahu villages are settled along the hill and border areas of Thailand-Myanmar and Thailand-Laos; therefore, it is not difficult for villagers to access MA.

Childhood experiences are widely studied in various populations in different aspects, including health problems [20]. In 2014, a study in China reported that some childhood experiences were associated with MA use in adulthood [21]. A study in the United States also reported that childhood experience and household dysfunction were associated with many health problems at later ages, including death in adults [22]. People begin using MA for different purposes, such as to have fun, to get more energy to work or to be accepted by peers [23]. Akha and Lahu youths begin using MA for several reasons, such as persuasion from their peers and level of access drugs [24]. However, there is no study detecting the associations between childhood experiences and MA use, particularly in hill tribe youths in Thailand, who are recognized as one of the most vulnerable populations for MA use.

Therefore, the study aimed to estimate the prevalence of MA use and determine the association of childhood experience with MA use among Akha and Lahu youths aged 15–24 years who lived in northern Thailand. The findings could be used to develop public health interventions for reducing MA use among Akha and Lahu hill tribe youths in Thailand.

## Methods

### Study design

A cross-sectional study design was applied to collect data from the selected participants.

### Study population

The study population included Akha and Lahu youths aged 15–24 years who lived in Chiang Rai Province, Thailand, in 2019.

### Study sample

The study sample was composed of Akha and Lahu youths aged 15–24 years who lived in Chiang Rai Province in 2019 and were randomly selected for the study. However, those who could not identify themselves as members of the Akha or Lahu tribes were excluded from the study. Moreover, both participants and parents who could not provide essential information regarding the study protocols were also excluded from the study.

### Sample size

The sample size was calculated by the standard formula for a cross-sectional study design [25]. After the calculation, based on the assumption of p = 0.27 [26], q = 0.73, and e = 0.05, there were 670 participants required for the analysis: approximately 335 for the Lahu tribe and another 335 for the Akha tribe.

Since, there is no scientific data available on the prevalence of the MA use among the hill tribe population, then, the calculation for the sample size was based on the information (prevalence) from the study conducted in Thai youth who lived in the central of Bangkok which was conducted by Toeam, et al [26]. Moreover, based on the information of the number of populations between the Akha and Lahu which was reported by the Hill tribe Welfare and Development Center [18], two tribes had similar size of the population living in 243 Akah villages (approximately 60,000 population) and 216 Lahu villages (approximately 50,000 population).

### Steps of data collection

In 2018, there were 243 Akha villages and 216 Lahu villages in Chiang Rai Province. Ten villages from each tribe were randomly selected by a simple random method. Government officers who were responding to the selected villages were asked for approval to perform the study in the targeted villages. After obtaining approval for access to the villages from the district officers, cooperation from the village headmen was obtained before collecting data. The lists of youths aged 15–24 years in the selected villages were obtained from the village headmen. All eligible individuals according to the lists received from the village headman were invited to participate in the study: 496 people from 10 Akha villages and 518 people from 10 Lahu villages. Appointments were made five days before assessing the participants to complete the questionnaire.

All participants were provided all information regarding the study protocols, particularly the security of the information obtained from all participants. Informed consent was obtained before completion of the questionnaire. Questionnaires were packed and sealed before being provided to the participants. Questionnaires were completed by participants in a personal private place and returned to researchers the next day. All questions in part one, two, four, five, and six were completed by participants (children) including part of socio-economic status of the family (part two), and nobody knew the content before reaching to researcher. However, questions in part three were separated and completed by their parents. A few people could not completely use Thai, then they were interviewed by researcher to complete the questionnaire. The reason to collect the information on experience of violence during individual’s aged 0–5 years, from parents was to improve the quality of the information. This protocol was proved from the pilot phase. The process of completion of the questionnaire was blinded, and no information could be referred back to any individual. The questionnaires were sent back to the researcher on the next day and completely sealed. All questionnaires were destroyed properly after coding. Data file was kept with security code.

### Research instruments

The questionnaire was developed based on the literature and discussion with five experts who were working in the fields of youth and child behaviors (3 people) and behaviors related to MA use (2 people) including the findings from our previous study [24]. The questionnaire consisted of six parts. In part one, 10 questions were used to collect data on general information such as age, sex, tribe, marital status, etc. In part two, 16 questions were used to collect information on the family, such as the relationship of the parents, number of family members, monthly family income, etc. In part three, 11 questions were used to collect an individual’s experience from 0–5 years of age, including abuse experience, such as history of assault and abuse from family members, abuse from peers in school, and sexual abuse. In this section, all information was obtained from the parents. In part four, 13 questions were used to collect information on history of being assaulted or abused while aged 6–14 years, including a history of school expulsion, assault by family members, assault by their peers, etc. In part five, 26 questions were used to collect information on personal behaviors such as smoking behavior, alcohol use, amphetamine use, etc. In part six, 20 questions were used to collect information on knowledge and attitude toward MA use. At the end of questionnaire, it appeared a short question on asking the experience in use of MA.

Subsequently, the questionnaire was examined for content validity by the item-objective congruence (IOC) technique, which was executed by three external experts in relevant fields: public health, psychology, and psychiatry. The feasibility and reliability of the questionnaire were detected by piloting with 10 selected Akha youths (5 males and 5 females) and another 10 selected Lahu youths (5 males and 5 females). The questionnaires were conducted three (3) times in the same piloting samples before being ready for use in the field. The sequencing and appropriateness of the questions were tested in the first and second rounds of the pilot. The last round was aimed at testing the reliability, which was found to have a Cronbach’s alpha of 0.78. The process of filling the questionnaire lasted 25 minutes for youth and 10 minutes for parents.

### Statistical analysis

Data were coded and double entered into an Excel file. Data files were transferred into SPSS version 24 (SPSS, Chicago, IL) for analysis. Descriptive data analysis was performed; categorical data were described in percentages. The means and its standard deviations (SDs) were used to describe the characteristics of continuous data. Logistic regression was used to detect the associations of childhood experiences with MA use among the Akha and Lahu youths at the significance level of alpha 0.05. The “Enter” mode was used in the step of selection independent variables into the statistical model. The pseudo R^2^ of Cox-Snell R^2^ and Nagelkerke R^2^, and the Hosmer- Lemshow chi-square were used to determine the fit of the model in all steps. Some variables were controlled the effect in the model which were determined as the confounder factors for the prediction. In the final model, all significant variables and controlled variables were fitted before making interpretations.

### Ethical approval and consent to participate

All research concept, procedures, and instruments were approved by the Mae Fah Luang University Research Ethic Committee on Human Research (REH-60141). Participants were asked their wiliness to participate the study by obtaining written informed consent form before completion the questionnaire in a private and confident room. Among those participants aged less than 18 years, parents were asked to agree in providing information in the questionnaire on behalf of their children by signing on the informed consent.

## Results

The participation rate was 77.6% (385 out of 496) in Akha, and 62.7% (325 out of 518) in Lahu. A total of 710 participants participated in the study; 54.2% were Akha, 52.5% were females, 50.6% were aged 15–17 years (mean = 18.1, SD = 2.7), and 11.4% did not have Thai ID cards. The majority were single (91.8%) and Christian (60.1%). Most participants had a high school and lower education (84.8%), lived with their parents (63.7%), and had 4–6 family members (66.9%) (Table 1).

**Table 1:** General characteristics of the participants.

Characteristics	Total n (%)	Akha n (%)	Lahu n (%)
**Total**	710 (100.0)	385 (54.2)	325 (45.8)
**Sex**			
Male	337 (47.5)	190 (49.4)	147 (45.2)
Female	373 (52.5)	195 (50.6)	178 (54.8)
**Age** (years)			
15–17	359(50.5)	208 (54.0)	151 (46.5)
18–20	216 (30.4)	119 (30.9)	97 (29.8)
21–24	135 (19.0)	58 (15.1)	77 (23.7)
Mean = 18.04, SD = 2.67			
**Marital status**			
Single	652(91.8)	372 (96.6)	280 (86.2)
Married	55(7.5)	12 (3.1)	43 (13.2)
Other	3(0.3)	1 (0.3)	2 (0.6)
**Religion**			
Buddhist	283(39.9)	128 (33.2)	155 (47.7)
Christian	427(60.1)	257 (66.8)	170 (52.3)
**Education**			
No educated	71(10.0)	22 (5.7)	49 (15.1)
Primary school	76(10.7)	18 (4.7)	58 (17.8)
Secondary school	163(23.0)	87 (22.6)	76 (23.4)
High school	292(41.1)	209 (54.3)	83 (25.5)
Vocational and university	108(15.2)	49 (12.8)	59 (18.2)
**Occupation**			
Student	448(63.1)	288 (74.8)	160 (49.2)
Employed	126(17.7)	50 (12.9)	76 (23.4)
Agriculturist	17(2.4)	8 (2.1)	9 (2.8)
Unemployed	119(16.8)	39 (10.1)	80 (24.6)
**Thai identification** (ID) **card**			
Yes	629(88.6)	339 (88.1)	290 (89.2)
No	81(11.4)	46 (11.9)	35 (10.8)
**Village location**			
Rural	352(49.6)	176 (45.7)	176 (54.2)
Semi-urban	358(50.4)	209 (54.3)	149 (45.8)
**Living with**			
Parents	452(63.7)	241 (62.6)	211 (64.9)
Father	49(6.9)	29 (7.5)	20 (6.2)
Mother	86(12.1)	59 (15.3)	87 (8.3)
Stepfather or stepmother	19(2.7)	16 (4.2)	3 (0.9)
Relatives	104(14.6)	40 (10.4)	64 (19.7)
**Parents’ status**			
Married and living together	510(71.8)	272 (70.6)	238 (73.2)
Either father or mother died	61(8.6)	38 (9.9)	23 (7.1)
Both father and mother died	13(1.8)	6 (1.5)	7 (2.2)
Separated	60(8.5)	38 (9.9)	22 (6.7)
Divorced	66(9.3)	31 (8.1)	35 (10.8)
**Number of family members** (people)			
≤ 3	119(16.8)	65 (16.9)	54 (16.6)
4–6	475(66.9)	246 (63.9)	229 (70.5)
≥ 7	116(16.3)	74 (19.2)	42 (12.9)
**Family income per month** (baht)			
≤ 10,000	213(30.0)	104 (27.0)	109 (33.5)
10,001–20,000	44(6.2)	26 (6.8)	18 (5.5)
≥ 20,001	44(6.2)	32 (8.3)	12 (3.7)
Unknown	409(57.6)	223 (57.9)	186 (57.3)

More than half of the participants had a family member who smoked (52.4%) and used alcohol (56.3%), while a few participants had a family member who used other substances. In the comparison analysis in experiences of family members on exposing to drugs and alcohol use between two tribes, it was found that no variable was found statistical significance (Table 2).

**Table 2:** Experiences of family members on exposing to drugs and alcohol.

Exposure	Total		Akha		Lah		χ^2^ *(p-value)*
	n	%	n	%	n	%	
**Having family member who ever smoked**							
No	338	47.6	191	49.6	147	45.2	1.36 *(0*.*244)*
Yes	372	52.4	194	50.4	178	54.8	
**Having family member who ever used alcohol**							
No	310	43.7	158	41.0	152	46.8	2.35 *(0*.*125)*
Yes	400	56.3	227	59.0	173	53.2	
**Having family member who ever used glue**							
No	690	97.2	378	98.2	312	96.0	3.07 *(0*.*080)*
Yes	20	2.8	7	1.8	13	4.0	
**Having family member who ever used methamphetamine**							
No	684	96.3	371	96.4	313	96.3	0.02 *(0*.*968)*
Yes	26	3.7	14	3.6	12	3.7	
**Having family member who ever used heroin**							
No	696	98.0	377	97.9	319	98.2	0.05 *(0*.*825)*
Yes	14	2.0	8	2.1	6	1.8	
**Having family member who ever used opium**							
No	687	96.8	376	97.7	311	95.7	2.18 *(0*.*140)*
Yes	23	3.2	9	2.3	14	4.3	

*Significant level at α = 0.05

The majority of caregivers while the participants were aged 0–5 years were mothers (72.4%), and most of the participants were supported by their family (58.2%). A few people had accidents (16.8%) and were hospitalized (29.4%) due to a health problem. Eighty-seven participants (12.3%) were assaulted by family members, and 15.6% were assaulted by peers in school. While having a comparison between tribes in the potential exposures relevant to MA use while aged 0–5 years, four variable were found the statistical differences; main care giver (p-value = 0.014), having accident (p-value = 0.035), having been hospitalized (p-value = 0.015), and having been physically assaulted by peer in school (p-value = 0.025) (Table 3).

**Table 3:** Potential exposures relevant to MA use while aged 0–5 years.

Family information	Total		Akha		Lahu		χ^2^ *(p-value)*
	n	%	n	%	n	%	
**Main caregiver**							
Mother	514	72.4	268	69.6	246	75.7	14.34 *(0*.014*)
Father	87	12.3	62	16.1	25	7.7	
Stepfather	10	1.4	3	0.8	7	2.2	
Stepmother	11	1.5	7	1.8	4	1.2	
Other relative	88	12.4	45	11.7	43	13.2	
**Used to be greatly supported by parents in regard to receiving desired food and beverages**							
No	644	90.7	354	91.9	290	89.2	1.54 *(0*.*214)*
Yes	66	9.3	31	8.1	35	10.8	
**Used to travel to desired places with support from parents**							
No	556	78.3	306	79.5	250	76.9	0.68 *(0*.*410)*
Yes	154	21.7	79	20.5	75	23.1	
**Used to be greatly supported by parents in regard to desired clothes and other items**							
No	610	85.9	339	88.1	271	83.4	3.17 *(0*.*075)*
Yes	100	14.1	46	11.9	54	16.6	
**Accident**							
No	591	83.2	310	80.5	281	86.5	4.46 *(0*.035*)
Yes	119	16.8	75	19.5	44	13.5	
**Hospitalization**							
No	501	70.6	257	66.8	244	75.1	5.88 *(0*.015*)
Yes	209	29.4	128	33.2	81	24.9	
**Head injury**							
No	600	84.5	326	84.7	274	84.3	0.02 *(0*.*893)*
Yes	110	15.5	59	15.3	51	15.7	
**Physically assaulted by family member**							
No	623	87.7	337	87.5	286	88.0	0.04 *(0*.*850)*
Yes	87	12.3	48	12.5	39	12.0	
**Physically assaulted by peer in school**							
No	599	84.4	314	81.6	285	87.7	5.03 *(0*.025*)
Yes	111	15.6	71	18.4	40	12.3	

*Significant level at α = 0.05

While participants were in the age range of 6–14 years, 65.4% were cared for by their mother, and few people had been supported by their family in regard to getting desirable food (5.2%) and travelling to desirable places (8.2%). Almost one-third had a head injury (16.2%). A few people were assaulted by family members (8.5%), assaulted due to their sexual orientation (4.1%), assaulted due to their socioeconomic status by their peers in school (12.1%), and sexually abused (1.3%). In the comparison analysis between tribes in the aspect of having the potential exposures relevant to MA use while aged 6–14 years, three (3) variable were found the statistical differences; having had travelled to desired places with support from parents (p-value = 0.02), had been greatly supported by parents in regards to desired clothes and other items (p-value = 0.048), and failed class examination (p-value = 0.010) (Table 4).

**Table 4:** Potential exposures relevant to MA use while aged 6–14 years.

Exposure	Total		Akha		Lahu		χ^2^ *(p-value)*
	n	%	n	%	n	%	
**Main caregiver**							
Mother	464	65.4	250	64.9	214	65.8	6.25 *(0*.*283)*
Father	113	15.9	70	18.2	43	13.2	
Stepfather	7	1.0	2	0.5	5	1.5	
Stepmother	15	2.1	8	2.1	7	2.2	
Relatives	111	15.6	55	14.3	56	17.3	
**Used to be greatly supported by parents in regard to receiving desired food and beverages**							
No	673	94.8	367	95.3	306	94.2	0.49 *(0*.*484)*
Yes	37	5.2	18	4.7	19	5.8	
**Travelled to desired places with support from parents**							
No	652	91.8	365	94.8	287	88.3	9.92 *(0*.002*)
Yes	58	8.2	20	5.2	38	11.7	
**Had been greatly supported by parents in regard to desired clothes and other items**							
No	45	6.3	367	95.3	298	91.7	3.92 *(0*.048*)
Yes	665	93.7	18	4.9	27	8.3	
**Accident**							
No	578	81.4	311	80.8	267	82.2	0.22 *(0*.*639)*
Yes	132	18.6	74	19.2	58	17.8	
**Hospitalization**							
No	557	78.5	293	76.1	264	81.2	2.74 *(0*.*098)*
Yes	153	21.5	92	23.9	61	18.8	
**Head injury**							
No	595	83.8	315	81.8	280	86.2	2.44 *(0*.*118)*
Yes	115	16.2	70	18.2	45	13.8	
**Expulsion from school**							
No	693	97.6	374	97.1	319	98.2	0.77 *(0*.*380)*
Yes	17	2.4	11	2.9	6	1.8	
**Assaulted by family member**							
No	650	91.5	354	91.9	296	91.1	0.17 *(0*.*678)*
Yes	60	8.5	31	8.1	29	8.9	
**Assaulted by peer in school**							
No	622	87.6	330	85.7	292	89.8	2.77 *(0*.*096)*
Yes	88	12.4	55	14.3	33	10.2	
**Insulted due to sexual orientation**							
No	681	95.9	372	96.6	309	95.1	1.08 *(0*.*300)*
Yes	29	4.1	13	3.4	16	4.9	
**Insulted due to socioeconomic status**							
No	624	87.9	339	88.1	285	87.7	0.02 *(0*.*884)*
Yes	86	12.1	46	11.9	40	12.3	
**Sexually abused**							
No	697	98.2	379	98.4	318	97.8	0.35 *(0*.*555)*
Yes	13	1.8	6	1.6	7	2.2	
**Failed class examination**							
No	420	59.2	211	54.8	209	64.3	6.59 *(0*.010*)
Yes	290	40.8	174	45.2	116	35.7	

*Significant level at α = 0.05

One hundred and three participants (14.5%) reported that they had used MA at least once in their life, 18.5% smoked, and 36.1% used alcohol. Most participants used Facebook (93.5%) and the Line application (73.8%). More than one-fourth (25.9%) had their urine tested for MA by police officer, and 7.6% had been arrested. Most participants had ≤5 close friends (78.6%), and of those close friends; 18.0% smoked, 27.5% used alcohol, and 2.1% used MA. The majority had an active and talkative personality (63.2%), were social (81.3%), and had high self-confidence (70.4%). While in the comparisons between tribes in behaviors and personalities, twelve (12) variables were found the statistical differences; regularly exercise (p-value = 0.001), ever played online games (p-value = 0.001), ever used Facebook (p-value = 0.001), frequency of Facebook use (p-value = 0.001), ever used the Line application (p-value = 0.004), ever tested for MA in urine by police officer (p-value<0.001), number of close friends (p-value<0.001), close friend who drink alcohol (p-value = 0.039), close friend who uses MA (p-value = 0.030), personality (p-value = 0.017), highly self-confident behavior (p-value = 0.014), and socialized behavior (p-value = 0.032) (Table 5).

**Table 5:** Participants’ behaviors and personality.

Characteristics	Total		Akha		Lahu		χ^2^*(p-value)*
	n	%	n	%	n	%	
**Ever used MA at least once**							
No	607	85.5	330	85.7	277	85.2	0.03 *(0*.*855)*
Yes	103	14.5	55	14.3	48	14.8	
**Ever smoked**							
No	579	81.5	307	79.7	272	83.7	1.83 *(0*.*176)*
Yes	131	18.5	78	20.3	53	16.3	
**Ever used alcohol**							
No	454	63.9	235	61.0	219	67.4	3.08 *(0*.*079)*
Yes	256	36.1	150	39.0	106	32.6	
**Regularly exercise**							
No	90	12.7	29	7.5	61	18.8	20.10 *(0*.001*)
Yes	620	87.3	356	92.5	264	81.2	
**Ever played online games**							
No	298	42.0	138	35.8	160	49.2	12.97 *(0*.001*)
Yes	412	58.0	247	64.8	165	50.8	
**Ever used Facebook**							
No	46	6.5	13	3.4	33	10.2	13.36 *(0*.001*)
Yes	664	93.5	372	96.6	292	89.8	
**Frequency of Facebook use** (n = 664)							
Sometimes	145	21.8	58	15.6	87	29.8	24.73 *(0*.001*)
Often	203	30.6	110	29.6	93	31.8	
Everyday	316	47.6	204	54.8	112	38.4	
**Ever used the Line Application**							
No	186	26.2	84	21.8	102	31.4	8.34 *(0*.004*)
Yes	524	73.8	301	78.2	223	68.6	
**Frequency of use of the Line Application** (n = 524)							
Sometimes	202	38.5	117	38.9	85	38.1	1.87 *(0*.*392)*
Often	175	33.4	94	31.2	81	36.3	
Everyday	147	28.1	90	29.9	57	25.6	
**Experienced a broken heart**							
No	339	47.7	181	47.0	158	48.6	0.18 *(0*.*670)*
Yes	371	52.3	204	53.0	167	51.4	
**Used to work in the night-work sector**							
No	688	96.9	375	97.4	313	96.3	0.70 *(0*.*402)*
Yes	22	3.1	10	2.6	12	3.7	
**Used to have sex in exchange for items or money**							
No	700	98.6	377	97.9	323	99.4	2.72 *(0*.*099)*
Yes	10	1.4	8	2.1	2	0.6	
**Ever tested for MA in urine by police officer**							
No	526	74.1	257	66.8	269	82.8	23.55 *(<0*.001*)
Yes	184	25.9	128	33.2	56	17.2	
**Arrested**							
No	656	92.4	357	92.7	299	92.0	0.13 *(0*.*716)*
Yes	54	7.6	28	7.3	26	8.0	
**Number of close friends** (people)							
≤ 5	558	78.6	280	72.7	278	85.5	17.33 *(<0*.001*)
6–10	141	19.9	98	25.5	43	13.2	
≥ 11	11	1.5	7	1.8	4	1.2	
**Close friend who smokes**							
No	582	82.0	306	79.5	276	84.9	3.53 *(0*.*060)*
Yes	128	18.0	79	20.5	49	15.1	
**Close friend who drinks alcohol**							
No	515	72.5	267	69.4	248	76.3	4.28 *(0*.039*)
Yes	195	27.5	118	30.6	77	23.7	
**Close friend who uses glue**							
No	695	97.9	379	98.4	316	97.2	1.25 *(0*.*264)*
Yes	15	2.1	6	1.6	9	2.8	
**Close friend who uses heroin**							
No	701	98.7	382	99.2	319	98.2	1.60 *(0*.*205)*
Yes	9	1.3	3	0.8	6	1.8	
**Close friend who uses MA**							
No	695	97.9	381	99.0	314	96.6	4.69 *(0*.030*)
Yes	15	2.1	4	1.0	11	3.4	
**Personality**							
Polite and quiet	200	28.2	100	26.0	100	30.8	8.20 (0.017*)
Active and talkative	449	63.2	260	67.5	189	58.2	
Stay alone	61	8.6	25	6.5	36	11.1	
**Highly self-confident behavior**							
No	210	29.6	99	25.7	111	34.2	6.03 (0.014*)
Yes	500	70.4	286	74.3	214	65.8	
**Socialized behavior**							
No	133	18.7	61	15.8	72	22.2	4.61 *(0*.032*)
Yes	577	81.3	324	84.2	253	77.8	

* Significant level at α = 0.05

In the univariate analysis that was performed to identify factors associated with MA use among the Akha and Lahu hill tribe youths, there were several factors associated with MA use, such as sex, age, occupation, parents’ marital status, number of family members, family member smoking status, family member alcohol use, and family member amphetamine use (Table 6).

**Table 6:** Univariate and multivariate analyses of factors associated with MA use among Akha and Lahu youths.

Factor	MA use				Univariate analysis			Multivariate analysis		
	Yes		No		OR	95% CI	p-value	AOR	95% CI	p-value
	n	%	n	%						
**Total**	**103**	**14.5**	**607**	**85.5**	**N/A**	**N/A**	**N/A**	**N/A**	**N/A**	**N/A**
**Sex**										
Male	91	27.0	246	73.0	11.13	5.97–20.76	<0.001*	4.75	2.27–9.95	<0.001*
Female	12	3.2	361	96.8	1.00			1.00		
**Age** (years)										
15–17	35	9.7	324	90.3	1.00			1.00		
18–20	35	16.2	181	83.8	1.79	1.08–2.96	0.023*	1.90	0.98–3.69	0.059
21–24	33	24.4	102	75.6	3.00	1.77–5.06	<0.001*	2.51	1.11–5.71	0.028*
**Tribe**										
Akha	55	14.3	330	85.7						
Lahu	48	14.8	277	85.2	1.04	0.68–1.58	0.855			
**Marital status**										
Single	96	14.7	556	85.3	1.00					
Married	6	10.9	49	89.1	2.90	0.26–32.25	0.387			
Other	1	33.3	2	66.7	0.71	0.30–1.70	0.441			
**Religion**										
Buddhist	44	15.5	239	84.5	1.00					
Christian	59	13.8	368	86.2	0.87	0.57–1.33	0.522			
**Education**										
Non-educated	19	26.8	52	73.2	4.39	0.53–36.04	0.169			
Primary school	20	26.3	56	73.7	4.23	0.52–35.10	0.175			
Secondary school	21	12.9	142	87.1	1.78	0.22–14.36	0.591			
High school	36	12.3	256	87.7	1.69	0.21–13.37	0.620			
Vocational and university	7	6.5	101	93.5	1.00					
**Occupation**										
Student	37	8.3	411	91.7	1.00					
Employed	31	24.6	95	75.4	3.66	2.13–6.31	<0.001*			
Agriculturalist	4	23.5	13	76.5	3.42	1.03–10.98	0.040*			
Unemployed	31	26.1	88	73.9	3.91	2.30–6.65	<0.001*			
**Thai identification card**										
Yes	97	15.4	532	84.6	1.00					
No	6	7.4	75	92.6	2.28	0.97–5.38	0.060			
**Village location**										
Rural	45	12.8	307	87.2	1.00					
Semiurban	58	16.2	300	83.8	1.32	0.87–2.01	0.197			
**Parents’ marital status**										
Living together	57	11.2	453	88.8	1.00					
Either father or mother died	15	24.6	46	75.4	2.59	1.36–4.94	0.004*			
Both father and mother died	1	7.7	12	92.3	0.66	0.09–5.19	0.695			
Separated	16	26.7	44	73.3	2.89	1.53–5.45	0.001*			
Divorced	14	21.2	52	78.8	2.14	1.12–4.10	0.022*			
**Number of family members** (people)										
≤ 3	25	21.0	94	79.0	3.16	1.41–7.11	0.005*			
4–6	69	14.5	406	84.5	2.02	0.98–4.18	0.058			
≥ 7	9	7.8	107	92.2	1.00					
**Family income per month** (baht)										
≤ 10,000	37	17.4	176	82.6	1.00					
10,001–20,000	7	15.9	37	84.1	0.90	0.37–2.17	0.815			
≥ 20,001	6	13.6	38	86.4	0.75	0.30–1.01	0.547			
Unknown	53	13.0	356	87.0	0.71	0.45–1.12	0.139			
**Having a family member who smokes**										
No	30	8.9	308	91.1	1.00	1.59–3.95	<0.001*			
Yes	73	19.6	299	80.4	2.51					
**Having a family member who uses alcohol**										
No	34	11.0	276	89.0	1.00					
Yes	69	17.3	331	82.8	1.69	1.09–2.63	0.019*			
**Having a family member who uses glue**										
No	93	13.5	597	86.5	1.00					
Yes	10	50.0	10	50.0	6.42	2.60–15.84	<0.001*			
**Having family member who uses methamphetamine**										
No	89	13.0	595	87.0	1.00			1.00		
Yes	14	53.8	12	46.2	7.80	3.50–17.40	<0.001*	5.04	1.66–15.32	0.004
**Having a family member who uses heroin**										
No	98	14.1	598	85.9	1.00					
Yes	5	35.7	9	64.3	3.39	1.11–10.33	0.032*			
**Having a family member who uses opium**										
No	95	13.8	592	86.2	1.00					
Yes	8	34.8	15	65.2	3.32	1.37–8.05	0.008*			
**Main caregiver from the ages of 0–5 years**										
Mother	63	12.3	451	87.7	1.00					
Father	17	19.5	70	80.5	1.74	0.96–3.14	0.067			
Stepfather	4	40.0	6	60.0	4.77	1.31–17.38	0.018			
Stepmother	3	27.3	8	72.7	2.69	0.69–10.39	0.153			
Other relative	16	18.2	72	81.8	1.59	0.84–3.01	0.153			
**Used to be greatly supported in regard to receiving desirable food and beverage from parents while aged 0–5 years**										
No	11	16.7	55	83.3	1.00					
Yes	92	14.3	552	85.7	1.20	0.61–2.38	0.601			
**Used to be greatly supported by parents in regard to travelling to desirable places while aged 0–5 years**										
No	27	17.5	127	82.5	1.00					
Yes	76	13.7	480	86.3	1.34	0.83–2.17	0.230			
**Used to be greatly supported by parents in regard to clothes and other items while aged 0–5 years**										
No	23	23.0	77	77.0	1.00					
Yes	80	13.1	530	86.9	1.98	1.17–3.33	0.010*			
**Had accident while aged 0–5 years**										
No	75	12.7	516	87.3	1.00					
Yes	28	23.5	91	76.5	2.12	1.30–3.45	0.003*			
**Had been hospitalized while aged 0–5 years**										
No	72	14.4	429	85.6	1.00					
Yes	31	14.8	178	85.2	1.04	0.66–1.64	0.874			
**Had head injury while aged 0–5 years**										
No	83	13.8	517	86.2	1.00					
Yes	20	18.2	90	81.8	1.38	0.81–2.37	0.235			
**Had been physical assaulted by family member while aged 0–5 years**										
No	77	12.4	546	87.6	1.00			1.00		
Yes	26	29.9	61	70.1	3.02	1.80–5.07	<0.001*	2.29	1.02–5.21	0.045*
**Had been physical assaulted by peer in school while aged 0–5 years**										
No	77	12.9	522	87.1	1.00					
Yes	26	23.4	85	76.6	2.07	1.58–3.42	0.004*			
**Major caregiver while aged 6–14 years**										
Mother	55	11.9	409	88.1	1.00					
Father	20	17.7	93	82.3	1.60	0.91–2.80	0.100			
Stepfather	3	42.9	4	57.1	5.58	1.21–25.58	0.027*			
Stepmother	7	46.7	8	53.3	6.51	2.27–18.65	<0.001*			
Relatives	18	16.2	93	83.8	1.64	0.88–3.07	0.121			
**Used to be greatly supported in regard to receiving desirable food and beverage from parents while aged 6–14 years**										
No	8	21.6	29	78.4	1.00					
Yes	95	14.1	578	85.9	1.68	0.75–3.78	0.211			
**Had been greatly supported by parents to travel to desirable places while aged 6–14 years**										
No	15	25.9	43	74.1	1.00					
Yes	88	13.5	564	86.5	2.24	1.19–4.19	0.012*			
**Had been greatly supported by parents in regard to clothes and other items while aged 6–14 years**										
No	96	14.4	569	85.6	1.00					
Yes	7	15.6	38	84.4	1.09	0.47–2.52	0.837			
**Had accident while aged 6–14 years**										
No	79	13.7	499	86.3	1.00					
Yes	24	18.2	108	81.8	1.14	0.85–2.32	0.186			
**Had been hospitalized while aged 6–14 years**										
No	76	13.6	481	86.4	1.00					
Yes	27	17.6	126	82.4	1.36	0.84–2.19	0.214			
**Had head injury while aged 6–14 years**										
No	78	13.1	517	86.9	1.00					
Yes	25	21.7	90	78.3	1.84	1.11–3.05	0.017*			
**Had been expelled from school while aged 6–14 years**										
No	92	13.3	601	86.7	1.00					
Yes	11	64.7	6	35.3	11.98	4.33–33.17	<0.001*			
**Had been physically assaulted by family member while aged 6–14 years**										
No	80	12.3	570	87.7	1.00			1.00		
Yes	23	38.3	37	61.7	4.43	2.50–7.84	<0.001*	3.15	1.32–7.54	0.010*
**Had been physically assaulted by peer in school while aged 6–14 years**										
No	81	13.0	541	87.0	1.00					
Yes	22	25.0	66	75.0	2.23	1.30–3.81	0.003*			
**Had been insulted due to sexual orientation while aged 6–14 years**										
No	97	14.2	584	85.8	1.00					
Yes	6	20.7	23	79.3	1.57	0.62–3.96	0.338			
**Had been insulted due to socioeconomic status while aged 6–14 years**										
No	78	12.5	546	87.5	1.00					
Yes	25	29.1	61	70.9	2.87	1.70–4.84	<0.001*			
**Was sexually abused while aged 6–14 years**										
No	96	13.8	601	86.2	1.00					
Yes	7	53.8	6	46.2	7.30	2.40–22.20	<0.001*			
**Failed a class examination while aged 6–14 years**										
No	62	14.8	358	85.2	1.00	0.69–1.61	0.087			
Yes	41	14.1	249	85.9	1.05	0.69–1.61	0.087			
**Number of close friends** (people)										
≤ 5	77	13.8	481	86.2	1.00					
6–10	23	16.3	118	83.7	2.12	0.73–2.02	0.447			
≥ 11	3	27.3	8	72.7	2.23	0.61–9.02	0.216			
**Having a close friend who smokes**										
No	51	8.8	531	91.2	1.00					
Yes	52	40.6	76	59.4	7.12	4.52–11.23	<0.001*			
**Having a close friend who drinks alcohol**										
No	57	11.1	458	88.9	1.00			1.00		
Yes	46	23.6	149	76.4	2.48	1.61–3.81	<0.001*	2.42	1.24–4.72	0.009*
**Having a close friend who uses glue**										
No	96	13.8	599	86.2	1.00					
Yes	7	46.7	8	53.3	5.46	1.93–15.40	0.001*			
**Having a close friend who uses heroin**										
No	96	13.7	605	86.3	1.00					
Yes	7	77.8	2	22.2	22.06	4.52–107.75	<0.001*			
**Having a close friend who uses MA**										
No	88	12.7	607	87.3	1.00					
Yes	15	100.0		0.0	5.46	1.93–15.40	0.001*			
**Personality**										
Polite and quiet	25	4.0	601	96.0	1.00					
Active and talkative	54	90.0	6	10.0	0.96	0.58–1.59	0.865			
Stays alone	24	3.8	601	96.2	4.54	2.34–8.81	<0.001*			
**Highly self-confident personality**										
No	20	9.5	190	90.5	1.00			1.00		
Yes	83	16.6	417	83.4	1.89	1.23–3.17	0.016*	2.35	1.17–4.69	0.016*
**Plays online games**										
No	27	9.1	271	90.9	1.00					
Yes	76	18.4	336	81.6	2.27	1.42–3.62	0.001*			
**Exercise regularly**										
No	24	26.7	66	73.3	1.00					
Yes	79	12.7	541	87.3	0.40	0.24–0.68	0.001*			
**Smokes**										
No	34	5.9	545	94.1	1.00			1.00		
Yes	69	52.7	62	47.3	17.84	10.96–29.05	<0.001*	8.27	4.42–15.46	<0.001*
**Uses alcohol**										
No	20	4.4	434	95.6	1.00					
Yes	83	32.4	173	67.6	10.41	6.20–17.50	<0.001*			
**Used to use a “Facebook” application**										
No	3	6.5	43	93.5	1.00					
Yes	100	15.1	564	84.9	2.54	0.77–8.35	0.124			
**Used to use a “Facebook” application**										
No	26	7.7	313	92.3	1.00					
Yes	77	20.8	294	79.2	3.15	1.97–5.06	<0.001*			
**Used to work in a night-work sector**										
No	95	13.8	593	86.2	1.00					
Yes	8	36.4	14	63.6	3.57	1.46–8.73	0.005*			
**Used to have sex in exchange for items or money**										
No	99	14.1	601	85.9	1.00					
Yes	4	40.0	6	60.0	4.05	1.12–14.60	0.033*			
**Has been arrested**										
No	69	10.5	587	89.5	1.00					
Yes	34	63.0	20	37.0	14.46	7.89–26.51	<0.001*			
**Knowledge on the impacts of MA use**										
Low	45	17.7	209	82.3	3.55	0.82–15.35	0.089			
Moderate	56	13.3	365	86.7	2.53	0.59–10.84	0.120			
High	2	5.7	33	94.3	1.00					
**Attitude on the impacts of MA use**										
Low	48	22.7	163	77.3	4.42	2.02–9.68	<0.001*			
Moderate	47	12.7	324	87.3	2.18	1.00–4.74	0.005*			
High	8	6.3	120	93.8	1.00					

*Significance level α = 0.05

After controlling for tribe, marital status, religion, education, occupation, and having Thai ID card in the multivariate model, 8 variables were found to be associated with MA use among the Akha and Lahu youths in northern Thailand: sex, age, having a family member who used MA, having been physical assaulted by family member while aged 0–5 years, having been physical assaulted by family member while aged 6–14 years, having a close friend who drinks alcohol, having a highly confident personality, smoking.

Males had a 4.75-fold (95% CI = 2.27–9.95) greater chance of MA use than females. Participants aged 21–24 years had a 2.51-fold (95% CI = 1.11–5.71) greater chance of MA use than those aged 15–17 years. Those who had a family member who used MA had a 5.04-fold (95% CI = 1.66–15.32) greater chance of MA use than those who did not. Those who had been physically assaulted by a family member while aged 0–5 years had a 2.29-fold (95% CI = 1.02–5.21) greater chance of MA use than those who had not. Those who had been physically assaulted by a family member while aged 6–14 years had a 3.15-fold (95% CI = 1.32–7.54) greater chance of MA use than those who had not. Those who had a close friend who used alcohol had a 2.24-fold (95% CI = 1.24–4.72) greater chance of MA use than those who did not. Those who had a highly confident personality had a 2.35-fold (95% CI = 1.17–4.69) greater chance of MA use than those who did not, and those who smoked had a 8.27-fold (95% CI = 4.42–15.46) greater chance of MA use than those who did not (Table 6).

## Discussion

Among the Akh and Lahu hill tribe youths who are living in northern Thailand, there was a high prevalence (14.5%) of MA use. There were also several factors related to MA use including personal characteristics, personality, family member and peer behaviors, and childhood experiences. Being male, being older, smoking, and having a highly confident personality were risk factors for using MA. Those who had a family member who used MA and had a close friend who used alcohol had a greater risk of using MA than those who did not. Childhood experiences of physical assault by a family member while aged 0–5 or 6–14 years were also associated with MA use among Akha and Lahu youths aged 15–24 years.

The prevalence of MA use among the youths who were studying in a vocational school in northern Thailand [27] was reported as 8.8%. This shows that the prevalence of MA use among the hill tribe youths (14.5%) is greater than that among the youths who were living in northern Thailand. It was also greater than the prevalence of MA use reported in Cambodia (10.4%) [28]. A greater proportion of MA use among the Akha and Lahu youths while comparing with Thai population, it could be supported by a qualitative study presented that social norms and also other positive personal perceptions among the Akha and Lahu were acting as major contributors for MA use in these population [24].

In our study, it was found that males had a significantly greater risk of using MA than females, which is consistent with a study conducted in Myanmar, which reported that males had a greater prevalence of MA users than females [29]. However, Dluzen et al [30] and Rungnirundorn et al [31] reported that females were more likely to be MA users and significantly more likely to be MA-dependent than males. This might be because in the culture of the Akha and Lahu hill tribe people, males dominate all activities at the family and community levels; therefore, males could expose to and use MA more than females [24].

In this study, it was also found that people aged 18–20 years had a greater risk of using MA than the youngest Akha and Lahu youths. This could be because older youths have income from work, and they could afford to use MA. Moreover, older youths may have many more close friends from socializing, and the opportunities to begin using MA could be greater than those among younger youths. The World Health Organization (WHO), Thailand, reported that Thai youths experienced their first use of drugs before the age of 14 years [32]. A study in Malaysia in 2018 [33] also reported that the age of beginning MA use was 13 years, which supports our finding. A report from a national survey on drug use and health in the United States in 2015 also reported that the early age of MA use was 12 years [34]. However, a study in Australia in 2019 [35] reported that among youths in Australia, the first use of MA occurred at 20 years, which is different from our study.

Smoking was found to be associated with MA use among Akha and Lahu youth in Thailand. This finding was supported by a study in Thailand that reported that smoking was significantly associated with the initiation of MA use among youths [27]. A study in Morocco also reported that smoking behavior was associated with MA use among high school children [36]. Moreover, in a review of an epidemiologic study in 2016 [37], it was found that smoking behaviors were greatly associated with MA use. In a systematic review, it was presented that smoking was a major predictor for MA use in various age categories [38].

A highly self-confident personality was also found to be associated with MA use among Akha and Lahu youths in Thailand. This finding could be explained by the fact that those who have high confidence would have a chance to engage in a new experience in their life, particularly in the use of MA among the Akha and Lahu youths. Due to youths being in a stage of life in which they are very eager to know their environment along with access to MA and a low education, youths can become MA users. This concept is supported by studies conducted in Taiwan [39] and in the United States [40]. However, a study in Iran reported the idea that a highly self-confident personality type was a protective factor for MA use [41]. However, the Alcohol and Drug Abuse Institute (ADAI) reported that those who had low confidence had a greater risk of initiating MA use among American people [42].

Having a family member who uses MA was greatly significantly associated with MA use among Akha and Lahu youths in Thailand. This is supported by a study by Chomchoie et al. [24]. The systematic review study clearly showed an association between a family history of drug use and MS use among youths [38].

A study in the United States reported that physical abuse before 15 years of age was a key factor associated with MA use and MA-related violence [40]. Moreover, a study in Morocco reported that living with an unsecure family was associated with MA use among youths [36]. Peltzer et al. [43] demonstrated that being a victim of physical assault, particularly by family members, was associated with MA use among youths in Asia. In our study, it was found that those children had been physical assaulted either during age of 0–5 years or 6–14 years or both periods had a greater chance of MA use that those who did not. A total of 108 cases were reported in having physical assaulted by family member from whole participants; 87 cases reported on age of 0–5 years, and 60 cases were reported on aged of 6–14 years. Among the victims, 38 out of 109 cases (34.8%) had experienced on physical assaulted by family member in both periods.

This may be children in childhood need to get love and care from people living around them particularly from their parents and family members to grow strong both physical and mental health. Children who grew up with love and safe environment, it could motivate to get desired outcomes in later years of age such as not use MA [44, 45].

In our study, it was found that children those who had a close friend who used alcohol had a greater chance of MA use than those who did not. This is supported by a study in the United Stated which was reported that those children who had a close friend who used alcohol had a greater chance to initiate MA than those who did not significantly [46]. A longitudinal study in rural cities, Wester United States, it was found that those adolescents who had a close friend who used alcohol was associated with substance use especially MA [47]. Moreover, a qualitative study in Thailand was also reported that having close friend who used alcohol led children to initiate MA [48].

Some limitations have been found in the study. First, identifying those people who used MA was difficult because it is an illegal substance in Thailand; therefore, most people who are using MA would not identify themselves as MA users. However, with the method of no information being traced back to any individual after filling in the questionnaire and the double-check method used by public health volunteers in a community to identify participants who used MA, the information gathered on the outcome would be closely related to the actual outcome. Under the current Thai government policy, all villagers have to be identified and classified in regard to whether they are using MA or not by their peers and an anonymous method. Those who are using MA are asked to participate in MA treatment programs in villages. This program is now working particularly well in rural villages and is managed by the Ministry of Public Health and other stakeholders [49]. Second, in part three of the questionnaire, questions were used to collect individual experience information related to when the participants were aged 0–5 years, particularly information related to physical assault by family members. These questions were answered by their parents, and the outcomes were shown to have high accuracy in the pilot test. Finally, three participants provided incomplete questionnaires, and they were excluded from the analysis. This small proportion of missing data would not interfere with the interpretation of the information.

## Conclusion

The study clearly shows the strong associations between childhood experiences while aged 0–14 years and personal behaviors and MA use among Akha and Lahu youths of older age in northern Thailand. Compared with other groups, male sex, smoking, older age, having close friends who use alcohol, and having a family member who uses MA were associated with MA use. Moreover, those who experienced physical assault from family members while aged 0–14 years were likely to use MA at a later age. Integrated intervention programs are urgently needed to reduce MA use among Akha and Lahu youths in Thailand; these programs should focus on improving family relationships and male individuals, smokers, and people with a highly confident personality. Moreover, the implementation should be focused in regularly monitoring and prevention on the physical assaulted during childhood by family members. The practical guideline on basic action while facing a problem of the physical assaulted in children by family members for the community health volunteers should be developed and provided. Strong collaborations among relevant agencies, both government and nongovernment, within countries and between counties are needed to address this problem.

## Supporting information

S1 File Questionnaire used in the study. (PDF)

S2 File Data file of the study. (XLSX)
