# Supplementary material for: Associations of childhood experiences and methamphetamine use among Akha and Lahu hill tribe youths in northern Thailand: A cross-sectional study
Source: PLoS One. 2020 Jun 18;15(6):e0234923. doi: 10.1371/journal.pone.0234923 (PMC7302480; doi:10.1371/journal.pone.0234923)
Supplement: S1 File — (PDF) [file pone.0234923.s001.pdf]

Questionnaire

Part 1 General information

1. Sex ☐ Male ☐ Female
2. Age…………....……. years
3. Ethnics ☐ Akha ☐ Lahu
4. Marital status ☐ Single ☐ Married Age..............years ☐ Divorce ☐ Widow
5. Religion ☐ Buddhism ☐ Christian ☐ Islam
6. Education
   ☐ Non-education ☐ Primary school ☐ Secondary school
   ☐ High School ☐ Vocational school ☐ University degree
7. Occupation
   ☐ Student ☐ Trader ☐ Employed
   ☐ Agriculturist ☐ Unemployed
8. Income ☐ No (Move to question no.9) ☐ Yes
   8.1 Characteristics of income ☐ Daily amount......................................Baht
   Use for ☐ Personal ....Baht
   ☐ Family ....Baht
   ☐ Saving ....Baht
   ☐ Monthly
   ☐ Unpredictable
9. Thai ID card
   ☐ Yes ☐ No
   Status ☐ Non-Thai identification card (Pink card)
   ☐ Undocumented persons (White card)
   ☐ No cards in hand
10. Thai speaking skill ☐ No ☐ Poor ☐ Excellence
    Thai listening skill ☐ No ☐ Poor ☐ Excellence
    Thai reading skill ☐ No ☐ Poor ☐ Excellence
    Thai writing ☐ No ☐ Poor ☐ Excellence
11. Residency area ☐ Municipality ☐ rural

Part 2 Family information

1. Parents' status
   ☐ Married and living together ☐ Either father or mother died
   ☐ Both father and mother died ☐ Separated ☐ Divorced
2. Number of family members...................people
3. Family income..................................Baht/Month (☐ Unpredictable)
4. Family-owned land ☐ No ☐ Yes amount................. Hectare
5. Family-owned fridge ☐ No ☐ Yes amount................. fridge
6. Family-owned television ☐ No ☐ Yes amount................. television
7. Family-owned mobile phone ☐ No ☐ Yes amount................. phone
8. Family-owned motorcycle ☐ No ☐ Yes amount................. motorcycle
9. Family-owned car ☐ No ☐ Yes amount................. car
10. If you're uncomfortable, who is the first person think about?
    ☐ Father ☐ Mother ☐ Brother/Sister ☐ Uncle/Aunt
    ☐ Grandfather/mother ☐ Lover ☐ Friend ☐ Other (Identify)........................
11. How much do you get warmth from family?
    ☐ Low ☐ Medium ☐ High (Always)
12. When parents quarrel with one another in most cases, how did your father fix the problem?
    ☐ Physical abuse ☐ Argue ☐ Walk away ☐ Compromise
13. When parents quarrel with one another in most cases, how did your mother fix the problem?
    ☐ Physical abuse ☐ Argue ☐ Walk away ☐ Compromise
14. Having a family member who smokes
    ☐ No
    ☐ Yes (☐ Father☐ Mother ☐ Brother/Sister ☐ Uncle/Aunt ☐ Grandfather/mother)
15. Having a family member who uses alcohol
    ☐ No
    ☐ Yes (☐ Father☐ Mother ☐ Brother/Sister ☐ Uncle/Aunt ☐ Grandfather/mother)
16. Having a family member who uses glue

☐ No
☐ Yes (☐ Father☐ Mother ☐ Brother/Sister ☐ Uncle/Aunt ☐ Grandfather/mother)

17. Having a family member who uses methamphetamine
☐ No
☐ Yes (☐ Father☐ Mother ☐ Brother/Sister ☐ Uncle/Aunt ☐ Grandfather/mother)

18. Having a family member who uses heroin
☐ No
☐ Yes (☐ Father☐ Mother ☐ Brother/Sister ☐ Uncle/Aunt ☐ Grandfather/mother)

19. Having a family member who uses opium
☐ No
☐ Yes (☐ Father☐ Mother ☐ Brother/Sister ☐ Uncle/Aunt ☐ Grandfather/mother)

Part 3 Personal information during ages of 0-5 years

1. Main caregiver from ages of 0-5 years
☐ Father ☐ Mother ☐ Stepfather ☐ Stepmother ☐ Grandfather/mother ☐ Uncle/Aunt

2. Used to be greatly supported by parents in regard to receiving desirable food and beverage from parents while aged 0-5 years
☐ No ☐ Rarely ☐ Sometimes ☐ Always

3. Used to be greatly supported by parents in regard to traveling to desirable places while aged 0-5 years
☐ No ☐ Rarely ☐ Sometimes ☐ Always

4. Used to be greatly supported by parents in regard to clothes and other items while aged 0-5 years
☐ No ☐ Rarely ☐ Sometimes ☐ Always

5. Had accident while aged 0-5 years
☐ No ☐ Yes

6. Had been hospitalized while aged 0-5 years
☐ No ☐ Yes

7. Had head injury while aged 0-5 years
☐ No ☐ Yes

8. Had situations while aged 0-5 years

Event	No	Sometimes	Always
8.1. Had been physical assaulted by family member			
8.2. Had been physical assaulted by peer in school			

Part 4 Personal information during ages of 6-14 years

1. Main caregiver from ages of 6-14 years
☐ Father ☐ Mother ☐ Stepfather ☐ Stepmother ☐ Grandfather/mother
☐ Uncle/Aunt

2. Used to be greatly supported by parents in regard to receiving desirable food and beverage from parents while aged 6-14 years
☐ No ☐ Rarely ☐ Sometimes ☐ Always

3. Used to be greatly supported by parents in regard to traveling to desirable places while aged 6-14 years
☐ No ☐ Rarely ☐ Sometimes ☐ Always

4. Used to be greatly supported by parents in regard to clothes and other items while aged 6-14 years
☐ No ☐ Rarely ☐ Sometimes ☐ Always

5. During the aged of 6-14 years, what would to desire to be while you grow up?
☐ No idea
☐ Police ☐ Doctor ☐ teacher ☐ Actor ☐ Others

6. Currently, do you work on as your previous desirable?
☐ Yes ☐ No
Reason; ☐ No education ☐ Did not get the support from parents ☐ No money
☐ Pregnant ☐ Other

7. Had accident while aged 6-14 years
☐ No ☐ Yes

8. Had been hospitalized while aged 6-14 years
☐ No ☐ Yes

9. Had head injury while aged 6-14 years
☐ No ☐ Yes

10. Had situations while aged 6-14 years

Event	No	Sometimes	Always
10.1. Had been expelled from school			
10.2. Had been physically assaulted by family member			
10.3. Had been physically assaulted by peer in school			
10.4. Had been insulted due to sexual orientation			
10.5. Had been insulted due to socioeconomic status			
10.6. Had been humiliated in public			
10.7. Was sexually abused			
10.8. Failed a class examination			
10.9. Had been teased by friends			

Part 5 personal information

1. Living with
   ☐ Father/Mother ☐ Father ☐ Mother ☐ Stepfather
   ☐ Stepmother ☐ Grandfather/mother ☐ Uncle/Aunt ☐ Husband/Wife
2. Close friend
   ☐ No ☐ Yes amount............. people
   Close friend (Identify amount)
   Smokes.......................people/Drinks alcohol.............people
   Glue......................... people / Methamphetamine...................... people
   Heroin...................... people
3. Main reasons that stop students from studying (More than 1 choice)
   ☐ No money ☐ Lack interest in studies ☐ No Thai ID card
   ☐ I'm not good in studies ☐ Sacrifice for siblings ☐ Pregnant
   ☐ Considerate to parents ☐ Run away from home ☐ Other…...............................
4. What type of personality do I have? ☐ Polite/less talking ☐ Cheerful/talkative ☐ Being Alone
5. Behaviors

Behaviors	No	Sometimes	Always
5.1. High self-confident behavior			
5.2. Rude speech			
5.3. Like to visit friends' houses /Socialize			
5.4. Unruly			
5.5. Play online games			
5.6. Drink alcohol/Hangout			
5.7. Play sport			

6. Smoke ☐ No ☐ Ever ☐ Yes amount............. cigarettes/Day
7. Drink alcohol ☐ No ☐ Ever ☐ Yes amount............. drinks/Day
8. Bike motorcycle ☐ No (Move to section 10) ☐ Yes
9. Your own motorcycle
   ☐ No (Move to section 10) ☐ Yes 9.1 Modified ☐ No ☐ Yes
   9.2 Racing ☐ No ☐ Yes
10. Use Facebook☐ No ☐ Yes (☐ Rarely☐ Often ☐ Everyday)
11. Use Line ☐ No ☐ Yes (☐ Rarely☐ Often ☐ Everyday)
12. Use Facebook/ line (The most of purpose)
    ☐ Work☐ Information ☐ Chat ☐ Other............................
13. Trust the information you read from Facebook / line?
    ☐ No ☐ Little ☐ Reliable
14. Experienced a broken heart
    ☐ No (Move to section 15) ☐ Yes

14.1 Pain from a broken heart
☐ No ☐ Little ☐ Pain ☐ Very painful
14.2 Ways To overcome heartbreak
☐ Enjoy with family ☐ Enjoy with friends ☐ Alone
☐ Self-harm ☐ Suicide attempt ☐ Drinks alcohol
☐ Smoke ☐ Drugs ☐ Other...............................

15. Do you like wearing long-sleeved shirts? ☐ No ☐ Sometime ☐ Everyday
16. How often do you bathe in 1 week? ☐ Everyday ☐ Sometime
17. In 1 week, how many days did you hang out and return after 8 pm?
☐ No ☐ Some days ☐ Everyday
18. Do you work at night?
☐ No ☐ Yes (Occupations ☐ Singer/Musician ☐ Restaurant serving ☐ Trader
☐ Guard ☐ Other...
19. Used to have sex in exchange for items or money ☐ No ☐ Yes
20. Arrested ☐ No ☐ Yes
21. Tests for MA in urine by police officer ☐ No ☐ Yes
22. Role model ☐ No ☐ Yes
23. Currently, live life as a role model ☐ No ☐ Yes
24. Life Goals
☐ No ☐ Yes (☐ Continue studying ☐ Working ☐ Buy a house / Build a house
☐ Buying a new car ☐ Saving ☐ Other...
25. Have you ever seen an amphetamine addict in the village? ☐ No ☐ Yes
26. Personality characteristics of drug addicts (More than 1 choice)
☐ Normal ☐ Cool ☐ Rich ☐ Scary ☐ Aggressive
☐Life problems ☐ Other...
27. Training on methamphetamine knowledge ☐ No ☐ Yes
28. In past 1 year, Training on methamphetamine knowledge ☐ No ☐ Yes
29. People selling methamphetamine in village ☐ No ☐ Yes
30. Policy in resolving the methamphetamine in village ☐ No ☐ Yes
31. Your village leader was involved in solving amphetamine problems
☐ Low ☐ Moderate ☐ Good
32. Relationship of people in your village
Help together ☐ Low ☐ Moderate ☐ Good
Conflict ☐ Low ☐ Moderate ☐ Good

Part 6 Knowledge and attitude about methamphetamine

6.1 Knowledge

Questions	True	False	Don't know
1. (Methamphetamine) These stimulants are controlled in Schedule I			
2. It as a white powder, injecting			
3. Drugs that may cause sleepiness			
4. Urine screens are the most common method of drug testing			
5. Up to 3 years' imprisonment or a fine of 10,000 – 60,000 Baht for consumption			
6. Heroin is the most active amphetamines.			
7. The brain damage caused by amphetamine used			
8. Opium is a plant used to produce amphetamines			
9. Glue and thinner are amphetamines of inhalable toluene substances			
10. Amphetamine may be habit-forming, and this medicine is a drug of abuse			

6.2 Attitudes

ข้อคำถาม	Agree	Disagree	Not sure

1. Methamphetamine is a stimulant drug that can make you feel extra energized			
2. Using low dose of methamphetamine does not cause drug addiction			
3. methamphetamine can be used to treat pain			
4. People who consume methamphetamine are bad people			
5. After taking methamphetamine cause to stress relief			
6. Methamphetamine are a matter of teenagers, if they grow up, they can quit themselves			
7. Drinking, and smoking often lead to buy methamphetamine			
8. Used methamphetamine does not lack of self and studies			
9. Amphetamine such as methamphetamine can make white skin and thin			
10. Amphetamine helps socialization easier, acceptance among friends.			

Finally, have you ever used methamphetamine once in your life? ☐ **No** ☐ **Yes**
