# Supplementary material for: Associations of childhood experiences and methamphetamine use among Akha and Lahu hill tribe youths in northern Thailand: A cross-sectional study
Source: PLoS One. 2020 Jun 18;15(6):e0234923. doi: 10.1371/journal.pone.0234923 (PMC7302480; doi:10.1371/journal.pone.0234923)
Supplement: Attachment — Submitted filename: Reviewers Comments and Responses.docx [file pone.0234923.s004.docx]

Reviewer #2: Although the author respond to comments, it is unclear.
1.Sampling method was straified cluster sampling, thus sample size calculation needs adjustment for design effect.

: Thank you very much professor for such great comment. We have tried to calculate the sample size by putiing the effect size, however, it is still in around 700. Moreover, the from the CIs particularly in presentation the significant, there were presented with very narrow such as in sex; OR=4.75, 95%CI=2.27-9.95, and aged 21-24 years; OR=2.51, 95%CI=1.11-5.71.

: Moreover, this is a cross-sectional without any previous information particularly in the prevalence of MA use in this population, therefore, we have carefully discussed with two statisticians; one from Mahidol University and another one is my professor from Harvard School of Public Health, both them suggested that with the assumptions, it is good to use the samples obtained in the study.

: However, we will keep in mind this significant point from your comments for our next work. Thank you very much professor.

2.Reliability process is unusal (three rounds with the same 20 samples and calulated alpha from last round). Recall bias appeared. Alpha should be presented for individual section rather than overall.

: This is common while developing the quality of the tool. We can not calculate the the reliability in the first round because after finishing the first round, many points were changed and improved. It meat that we need to get the final and completed version to test and calculate for reliability score before use.

: Another very important point is this is the pioneer project on doing in the MA problem among the hill tribe who have limited in use Thai. Then, we had had very carefully obtained the information and also approached tham.

: I very hope that you understand us and thank you very much.
3. Table 2-5 should be compare between two hill-tribes.

: thank you for the comment, it’s revised and improved.

4. Although the MA lifetime prevalence is 3 times higher than general population of Thailand in 2019, it should be dicussed why it is. The specific culture of these hill-tribes affected to MA use still need to clarify.

: Thank you very much for the comment. It’s improved in page no.

Reviewer #4: The authors were responsive to comments and the paper is now improved and has greater clarity overall. However, not all the relevant responses that are given in the letter to the editor are included in the paper. I have a few remaining comments:

Specific comments:
1. In the abstract, conclusions: the last sentence and intervention for “male youth” need to be better explained (I suppose as a target population) – this sentence is still not well phrased. is the intention to say: …interventions that lowers risk of MA use by addressing family relationship, male youth risk behaviors….etc….?

: Thank you for comment, it’s improved.

2. Sample size calculation: still need clearer description of what was done under what assumption. if the calculations were to estimate the proportion of MA use, then a single sample with confidence interval widths could be used. if it is based on comparing two proportions (which is what is indicated), then, explain which groups are compared and if this is within each tribe group? - this may not be a critical point in the paper but if included, it should be clear and relevant to the analysis performed.

: Thank you so much for the valuable comment in this point. It has been revised an improved in section of sample size calculation.

: Since, there is no scientific data available on the prevalence of the MA use among the hill tribe population, then, the calculation for the sample size was based on the information (prevalence) from the study conducted in Thai youth who lived in the central of Bangkok which was conducted by Toeam, et al [26]. Moreover, based on the information of the number of population between the Akha and Lahu which was reported by the Hill tribe Welfare and Development Center [18], two tribes had similar size of the population living 243 Akah villages (approximately 60,000 population) and 216 Lahu villages (approximately 50,000 population).

3. As more than one youth per household could participate, can the authors add the info on how many clusters with size>1 were included? as the analysis does not take this clustering into account at a minimum, a note referring to potential biases in estimating the statistical significance (standard errors of estimates) should be included in methods and/or discussion. this is an important methodological issue.

: We have revised our raw data, and it was found that no household or family that presented more than one participant. We accept that we never though this issue before. We have learned this new point, thank you very much.

: However, we have put information in section of “step of data collection” to make clear the point.

4. Results: I do not find the reported “response rate” to the survey in the manuscript.

: We have responded to this point in our previous version regarding to the comment from one of reviewers. Please see the first sentences on the result section.

5. Table 1-5: the paper would benefit by creating tables 1-5 that contain more information including: a) the original n & 5 columns for overall frequencies and combining with it the univariate analysis that appears in table 6. then table 6 would just have the final multivariate table. this will be make it easier for the reader to capture the final model.

:Thank you very much for the comment. However, the previous reviewer suggested to put more statistics in table no.2-5 (Comment no.2). Therefore, we would like to maintain on univariate and multi variate analyses in same table (table 6). It’s also much more easier in explain the relationship between variables. I very hope that you understand us.

6. Description on how the MV model developed should be added to methods (it is in the response letter but not in the paper)

: Thank you very much for the comment. It’s improved in page no. 6

7. for goodness of fit for logistic mode – the c-statistics (or area under the ROC) is preferable.

: Thank you very much. This is one thing that I have learned thank you so much.

Thank you so much!

TK

Regards,

TK

Assistant Professor Dr.Tawatchai Apidechkul

Deputy Dean, School of Health Science, MFU

Director, Center of Excellence of the Hill tribe Health Research, WHO-CC

Former Hubert H Humphrey Fellow (2013-2014), Emory University

Global Health Delivery Intensive (Harvard School of Public Health)
