# Supplementary material for: Associations of childhood experiences and methamphetamine use among Akha and Lahu hill tribe youths in northern Thailand: A cross-sectional study
Source: PLoS One. 2020 Jun 18;15(6):e0234923. doi: 10.1371/journal.pone.0234923 (PMC7302480; doi:10.1371/journal.pone.0234923)
Supplement: Attachment — Submitted filename: Editor Comments and Response.docx [file pone.0234923.s005.docx]

Additional Editor Comments (if provided):

Thanks for your revisions. The manuscript is now much improved and almost ready for publication. However, since PLOS ONE does not allow you to proofread your manuscript after acceptance, I would like you to take this opportunity to do so. Here are some examples for your consideration:

: Thank you so much!

1. I am not sure if ‘Thai-Myanmar-Laos Republic’ border is correct. If the ‘Republic’ is for Laos, the more commonly used is ‘Lao People's Democratic Republic’ or just ‘Laos.’

: Thank you so much for the correction in the point, it’s replaced by “Laos” in whole tex.

2. You may consider using ‘adverse childhood experiences’ which is widely used in the literature instead of ‘bad childhood experiences.’

: As we have responded to the point in previous version that in this study that the cross-sectional was used to explored on all factors relevant to MA use in late years of the Akha and Lahu youths. Having bad experience while very early ages were determined as one of factors in the model. Therefore, we have decided that if it is used the word of “adverse childhood experience” may not reflect the study concept.

: However, we concern a lot and have had long discussion in the point.

: Thank you very much for your concern, and now we are planning a new project to detect a particular of impact of “adverse childhood experience” among these population by a stronger study design.

: Thank you once again for your valuable suggestion.

3. Abstract:
- First sentence in Methods may be revised to avoid repeating the objective.

: Thank you so much, it’s improved.

- Results: After controlling for…

: Thank you, it’s improved

- Since the analyses included both Akha and Lahu youth, repeating the expression ‘…among Akha and Lahu youth in northern Thailand’ brings more confusions than helps and unnecessarily increased the word count.

: Thank you, we agree with you and it’s improved.

- I am not sure if ‘ORadj’is commonly used. May consider ‘adjusted odds ratio (AOR).

: Replaces all

4. Methods:
- It would great if you could add a little bit more information on the inclusion criteria for both youth (participants) and parents. More information of the selection of the participants (youth and parents) is also required.

: It’s improved. Detail of selection the participants is provided in the “Step of data collection”

- Since this study was conducted among tribal populations, research instrument should also include information on the languages used for the questionnaire for each tribe, if translation (and back-translation) was performed, and average time required for the interview.

: Thank you for great comment, it‘s improved. In the section of language, the questionnaire is provided in Thai because all of the participants including their parents are able to use Thai.

- ‘The questionnaires were conducted three (3) times in the same piloting samples…’ This is hard to understand: what does this mean? How many questionnaires were developed? If different questionnaires were developed for youth and parents, this should be clearly described.

: Since this is the pioneer of the project relevant to the MA use in the hill tribe, therefore, no standard or other questionnaire are available.

In this step (pilot), we had several purposes to do this such as sequencing of the questions, words or sentences used weather agiant culture or belief, or feel stigma or not, questions make free of sense to answer or not. Therefore, it’s needed to have many times to repeat for making sure the quality of the questionnaire is met.

: The questions for children, were tested among the youths and the questions for the parents were asked their parents accordingly.

: We developed only one set of questionnaires, with having six parts, presented detail in page. 4-5

: We very hope you understand our situation.

- The flow of the information would be better if the sampling procedures (Steps of data collection) comes before ‘Research instruments.’

: Thank you, it’s re-sequence accordinly

- ‘All questionnaires were properly destroyed after coding’ – what does this really mean? After data entry?

: It means that all filled questionnaire form (hard copy by participants and parents, there were destroyed after coding and putting into the computer by cutting it into a very small piece and burning with the university waste management processing. This reflects on the comments of the Ethical Consideration Board that to protect the release of any information to public which we has strictly followed the comments.

- The writing of ‘Statistical analysis’ can be improved by removing typos and grammatical errors. ‘Conceptual framework’ was mentioned without earlier discussion.

: Thank you so much, it’s improved.

5. Please proofread the whole manuscript accordingly.

: We all (authors) have carefully looked throughout the whole paper at least three rounds and many points have been improved. Thank you so much.

Thank you so much!

TK
